# Report of Clinics at Atlanta Medical College*We intend in the future to report some of the Clinics that are held in the Colleges of this city. Our report in this issue is made somewhat brief from lack of time. Hereafter they will be more complete.

**Published:** 1896-02

**Authors:** Ralcey W. Bell

**Affiliations:** Atlanta Medical College


### REPORT OF CLINICS AT ATLANTA MEDICAL COLLEGE.*

By RALCEY W. BELL,

Atlanta Medical College.

EYE, EAR, NOSE, AND THROAT—(*Prof. A. W. Calhoun.*)

*January 10, 1896.*—Mrs. E. A., aged sixty-one, Parks, Ga. Senile cataract, mature in right eye, immature in the left eye. Graefe's extraction from right eye. Operation a perfect success. Patient doing well.

*January 16, 1896.*—Mr. D. A. McL., Inverness, Ga., aged twenty-eight. Pterygium of the left eye, of full growth, closely adherent, involving cornea. Complete removal and patient convalescent.

*January 16, 1896.*—Mrs. R., aged sixty-two, city. Dacryocystis, right eye. Successful operation and patient doing well.

*January 16, 1896.*—M. F., aged forty-three, city. Chalazion of left eye. Complete removal from beneath; rapid and complete recovery.

*We intend in the future to report some of the Clinics that are held in the Colleges of this city. Our report in this issue is made somewhat brief from lack of time. Hereafter they will be more complete.

*January 16, 1896.*—E. R., aged fifty-four, Villa Rica, Ga. Senile cataract, mature in right eye, immature in left eye. Graefe's extraction from the right eye; operation successful. Patient convalescing.

*January 16, 1896.*—F. M., aged sixteen, Stockbridge, Ga. Purulent ophthalmia in both eyes, followed by perforating ulcer of left eye, leaving scar.

Treatment:

℞ Zinci. sulph.,
Morph. sulph................................................āā gr. ij.
Alum. sulph................................................gr. iv.
Aquæ................................................℥ i.
M. Sig.: Drop in eyes three times daily.

Also,

℞ Hydrarg. oxid. flav................................................gr. ss.

Vaselin................................................ʒ j.

M. Sig.: Apply at night.

GYNECOLOGY—(*Prof. V. O. Hardon*).

*January 13, 1896.*—Mrs. L. T., aged twenty-three, cook, city. Patient complains of severe pain in abdomen with mild uterine hemorrhage during the past twenty-four hours; says she is three months pregnant; has borne two children, the younger being a year old. Gives no history of miscarriages; thinks she "strained herself." Examination shows pulsation of uterine artery, softening but no dilatation of cervix, slight discharge of blood and most of the presumptive signs of pregnancy more or less clearly marked. Uterine enlargement indicates middle of pregnancy. Diagnosis: Threatened miscarriage, probably due to overwork.

Treatment: Absolute rest, and

℞ McMunn's elix. opii................................................ʒ ijss.
Fl. ext. viburnum q.s. ad................................................℥ ij.
M. Sig.: Teaspoonful every two hours until pains cease.

*January 13, 1896.*—Mrs. J. B., aged forty, washerwoman, city. Patient complains of pain in breast and in region of the left ovary extending antero-posteriorly. Menstruation regular, during which there is no increase of pain. Examination shows typical case of carcinoma of cervix, involving the entire cervix, upper part of vaginal wall and broad ligament of the right side. Treatment: Local antiseptics and general systemic tonics.

*January 13, 1896.*—Mrs. M. T., aged twenty-one, washerwoman, city. Pain in uterus for the past year—miscarried four times and had one premature labor; menstruates irregularly—constant leucorrhea—abdomen tender to the touch, painful to walk, etc. Examination shows uterus in condition of subinvolution; ovaries enlarged and tender to the touch. Right ovary immovable. Treatment: Glycerine tampon, under which the patient shows decided improvement.

*January 13, 1896.*—Addie A., aged thirty, cook, city. Pain in region of the ovaries, which had been removed by Dr. Hardon four years ago. Diagnosis: Imprisoned nerve filaments in cicatricial tissue of the stumps of the ovaries with probable adhesions. Were the pain severe enough and should consensus of symptoms warrant it, the treatment would be to reopen the abdominal cavity and break up adhesions, etc. Under the circumstances and for the present, at least, the treatment will consist of hot local applications and tincture of the chloride of iron.

DERMATOLOGY AND SYPHILIS—(*Dr. M. B. Hutchins*).

Here is a case of *Tuberculosis Cutis Verrucosa:*

J. K., aged thirty-nine. Farmer, Nacoochee, Ga. Three years ago a "gathering" in outer anterior surface right leg near the middle. This was opened by a friend with his pocket knife. Ulcerative action dated from this. The disease gradually progressed outwards and inwards until the borders, first farthest apart, met over the inner side of the tibia. Smooth scar tissue marks the area passed over, about the middle two-fourths of the outer and posterior surfaces of the leg. The edges show a band of active disease about one inch wide, indolent, ulcerous, papillomatous, and crusted; odor offensive. An indolent crusted ulcer, with red areola, size of a silver dollar, in right Scarpa's triangle. On left buttock running to fold and tip of coccyx a patch of dry one-half pea-sized papular lesions, warty-looking, whole patch about six square inches.

[Crusts were removed from leg with salicylated oil and bathing. Patient declined treatment with Paqurlin, and returned to his home.]

